# PI-RADS v2.1 evaluation of prostate “nodule in nodule” variants: clinical, imaging, and pathological features

**DOI:** 10.1186/s13244-024-01651-6

**Published:** 2024-03-18

**Authors:** MingHua Sun, Li Xu, XiaoYan Zhang, LiYu Cao, WenBao Chen, Kai Liu, Hao Wu, DongDong Xie

**Affiliations:** 1 Department of Radiology, the Fuyang Hospital of Anhui Medical University, Fuyang, People’s Republic of China; 2 Department of Pathology, the Fuyang Hospital of Anhui Medical University, Fuyang, People’s Republic of China; 3 Department of Urology, the Fuyang Hospital of Anhui Medical University, Yingzhou District, No. 99, Mount Huangshan Road, Fuhe Modern Industrial Park, Fuyang, Anhui Province 236000 People’s Republic of China; 4 Medical Imaging Center, The Fuyang Tumor Hospital, Fuyang, People’s Republic of China

**Keywords:** Nodule in nodule, PI-RADS v2.1, Prostate biopsy, Prostate cancer, Prostate-specific antigen density

## Abstract

**Objectives:**

To analyze the correlation among the imaging features of prostate “nodule in nodule,” clinical prostate indices, and pathology results.

**Methods:**

We retrospectively analyzed the prostate images from 47 male patients who underwent MRI scans and pathological biopsy from January 2022 to July 2023. Two radiologists (R1/R2) evaluated the morphology and signal intensity of the “nodule in nodule” in a double-blind manner and calculated the PI-RADS v2.1 score, which was compared with clinical prostate indices and pathological results.

**Results:**

34.04% (16/47) of patients were pathologically diagnosed with clinically significant prostate cancer (csPCa). Total prostate-specific antigen (tPSA), free/t PSA, PSA density (PSAD), and prostate gland volume (PGV) were significantly different between csPCa patients and benign prostatic hyperplasia (BPH) patients with prostate “nodule in nodule”. R1/R2 detected 17/17 prostate “nodule in nodule” pathologically confirmed as csPCa on MRI; 10.60% (16/151) (R1) and 11.11% (17/153) (R2) had diffusion-weighted imaging (DWI) PI-RADS v2.1 score of 4, and 0.66% (1/151) (R1) had a score of 3. The percentages of encapsulated, circumscribed, and atypical nodules and obscured margins were 0.00% (0/151), 0.00% (0/151), 5.96% (9/151), and 5.30% (8/151), respectively, for R1, and 0.00% (0/153), 0.00% (0/153), 5.88% (9/153), and 4.58% (7/153) for R2.

**Conclusion:**

When the inner nodules of “nodule in nodule” lesions in PI-RADS v2.1 category 1 in the TZ show incomplete capsulation or obscured margins, they are considered atypical nodules and might be upgraded to PI-RADS v2.1 category 3 if they exhibit marked diffusion restriction. However, further validation is needed.

**Critical relevance statement:**

This study first analyzed the relationship between clinical and pathological findings and the size, margin, and multimodal MRI manifestations of the prostate “nodule in nodule.” These findings could improve the diagnostic accuracy of PI-RADS v2.1 for prostate lesions.

**Key points:**

• The margin of the prostate inner nodules affects the PI-RADS v2.1 score.

• The morphology of prostate “nodule in nodule” is related to their pathology.

• The PI-RADS v2.1 principle requires consideration of prostate “nodule in nodule” variants.

**Graphical Abstract:**

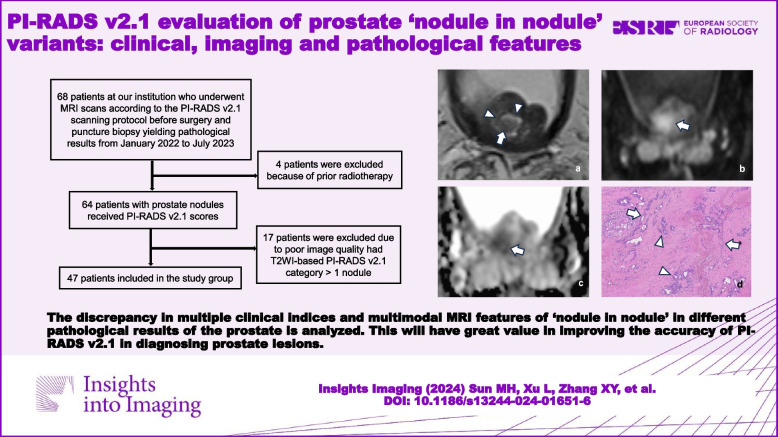

## Introduction

The Prostate Imaging and Reporting Data System (PI-RADS) is a standardized method based on multiparametric MRI (mpMRI) supervisor scoring for the semiquantitative evaluation of prostate lesions [1–3]. Although it has been clinically and pathologically proven to be a useful and effective evaluation tool [4, 5], its accuracy in grading clinically significant prostate cancer (csPCa) through targeted biopsy has been inconsistent across studies [6, 7]. Recent studies by Yilmaz EC et al. [8] have shown that the detection rates of csPCa among lesions with PI-RADS v2.1 scores of 1, 2, 3, 4, and 5 points are 0%, 9%, 14%, 37%, and 77%, respectively. Consequently, numerous researchers have investigated ways to improve the diagnostic accuracy of mpMRI for detecting csPCa and reducing unnecessary biopsy procedures [9, 10].

For nodules in the transition zone (TZ) with a score of 2–3 on T2-weighted imaging (T2WI), the PI-RADS v2.1 category was determined according to the score from diffusion-weighted imaging (DWI) [11]. In clinical practice, one or more small nodules are often found within prostate nodules [12]. There is a certain correlation between the integrity of the capsule of the inner nodule and pathology. However, whether other morphological features of the inner nodule, such as margin, shape, and signal intensity, also have similar effects has rarely been reported. The purpose of this study was to determine the correlation between the morphology and pathology (using targeted, ultrasound-guided, and systematic 12-core biopsy as a reference standard) of “nodule in nodule” among PI-RADS v2.1 category 1 lesions.

## Materials and methods

### Patients

**Fig. 1:**
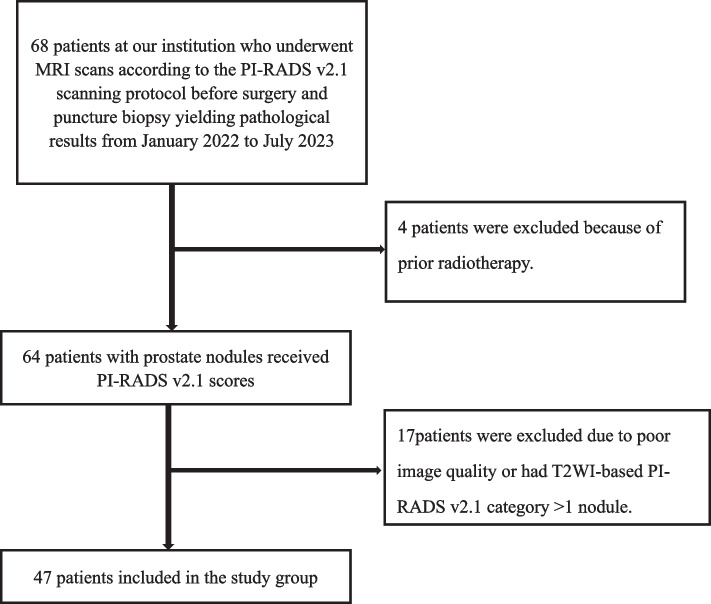
Flow diagram of patient selection used in this consecutive retrospective study

This retrospective study was approved by the local research ethics committee (Ethics Committee of Fuyang Hospital of Anhui Medical University, Fuyang, China. Code:KY2023063), and informed consent was obtained from the patients. We analyzed the data from an existing database of 68 patients at our institution in whom digital rectal examination (DRE) revealed suspicious nodules in the prostate gland or who had a PSA > 10 ng/mL and who underwent MRI scans according to the PI-RADS v2.1 scanning protocol followed by a pathological examination (from January 2022 to July 2023). We excluded 21 patients who had undergone prior radiotherapy and had poor image quality or had T2WI-based PI-RADS v2.1 scores > 1 nodule. Finally, 47 patients were included in the study. The flowchart for patient screening is shown in Fig. 1. The nodules determined by mpMRI scanning were biopsied through the rectum under ultrasound guidance by experienced urologists using the same method as described in previous studies [13, 14].

### MRI technique and image analysis

All mpMRI images were collected by a 3.0 T MRI scanner (Veta, Siemens Healthineers) with a pelvic surface coil and activated spine coils (4–16 channels and 8–12 channels, respectively). The scanning protocol is provided in Table 1.

**Table 1:** Protocol sequence parameters for multiparametric MRI of the prostate

	T1WI TSE	T2WI TSE			DWI	DCE
Imaging plane	Axial	Axial	Coronal	Sagittal	Axial	Axial
Field of view (FOV) (mm)	350 × 100	200 × 100	220 × 100	220 × 100	220 × 100	350 × 81.3
Matrix size	352 × 70	320 × 80	320 × 80	320 × 80	100 × 100	70 × 70
Slice thickness/gap (mm)	3.0	4.0/0.8	3.0	3.0	3.0/1.0	3.0
TR/TE (ms)	533.0/8.9	3410.0/101	5000.0/108	5000.0/108	5800/78	5.47/2.46
Flip angle	110	160	110	110		9.0
Receiver bandwidth (Hz/voxel)	200	200	274	274	1724	870
Acquisition time (min)	2:05 min	2:32 min	2:20 min	2:20 min	2:43 min	2:54 min
Number of signals averaged	1	3	1	1	4	
*b* value (s/mm^2^)	-	-	-	-	50, 800, 1200, 1500	-

Two urogenital radiologists trained in PI-RADS v2.1 (M.H.S. with 17 years and L.X. with 10 years of experience in interpreting mpMRI scans of the prostate) estimated the MRI-based imaging scores of prostate nodules separately and were blinded to the clinical and pathological data while knowing that all specimens had been obtained from biopsy. “Nodule in nodule” was defined as a nodule with a complete capsule containing a smaller nodule (i.e., a second-layer nodule) [12]. Referring to the PI-RADS v2.1 prostate sector map, the radiologists recorded the location, shape, margin [15], size along the long axis [12], and PI-RADS v2.1 category of the nodules. For the “nodule in nodule,” in addition to the description above, the PI-RADS v2.1 DWI score, category, and the ratio of the length to the diameter along the long axis of the inner and external nodules were also recorded. The prostate grand volume (PGV) was calculated from the 3-axis measurements using the ellipsoid formula [16]. It is defined as follows: ML (cm) × AP (cm) × CC (cm) × π/6.

### Reference standard

Prostate biopsy was performed by a team of urologists (D.D.X.) with more than 10 years of experience who had performed more than 200 targeted prostate biopsies in the prior 5 years using one of two biopsy devices (Aplio 500, Toshiba or HI VISION Preirus, Hitachi Medical Systems) under ultrasound guidance based on mpMRI scans, including targeted biopsy and systematic 12 + X core biopsy (using the puncture method described by Maggi M [17, 18]). All biopsy specimens were reviewed by a urogenital pathologist (L.Y.C.) with more than 25 years of experience in PCa pathology.

### Statistical analysis

The basic and clinical data of the patients, including the number of “nodule in nodule,” as well as the direct ratio in the long axis plane, were recorded. Statistical analysis was performed using the SPSS 23.0 statistical software. Cohen’s kappa test [19] was conducted on the subjective scoring results of the two radiologists. Intraclass correlation coefficient (ICC) consistency analysis [20] was performed on the objective measurement results of the two radiologists (where 0.75 indicates good consistency, 0.4-0.75 indicates average consistency, and < 0.4 indicates poor consistency). Single-factor ANOVA was used to compare the objective indicators between the groups, and *p* < 0.05 indicated a statistically significant difference. Measurement data are presented as the mean ± standard deviation (*x̄* ± *s*) and were compared between groups with the *t*-test.

## Results

**Fig. 2:**
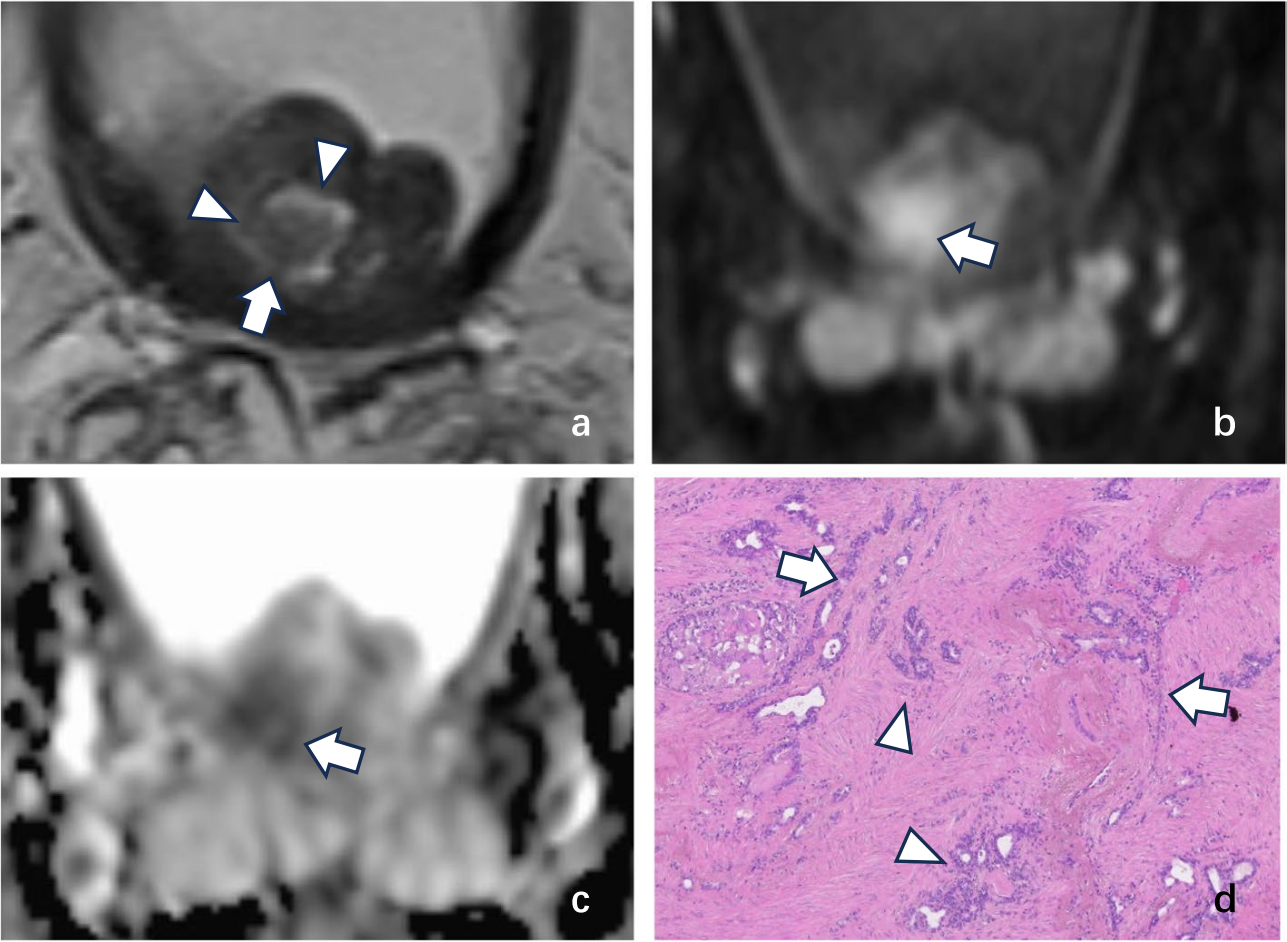
A 65-year-old man with a right transition zone “nodule in nodule” of PI-RADS v2.1 category 1 with cancer. **a** Axial T2W MR image demonstrating that the external nodule was completely encapsulated (arrowheads). The inner nodule (white arrow) was characterized by an obscure margin by both radiologists. On the (**b**) echo-planar DWI (*b* = 1500 mm.^2^/s) map and (**c**) ADC map, the inner nodule (white arrow) was characterized by marked restriction diffusion (DWI score of 4) by the two radiologists. **d** Prostate biopsy histopathological imaging of the “nodule in nodule” yielded a Gleason score 4 + 5 for prostate acinar cell carcinoma: Gleason grade 4 is characterized by glandular fusion and sieve-like, glomerular-like, or nonglandular luminal structures (arrowheads); the structure of the 5th grade glandular (arrow) cavity of the Gleason system has almost completely disappeared, with only occasional glandular cavities visible. The epithelial cells form solid patchy, linear structures, or infiltrate into the stroma as single cells (HE × 100)

**Fig. 3:**
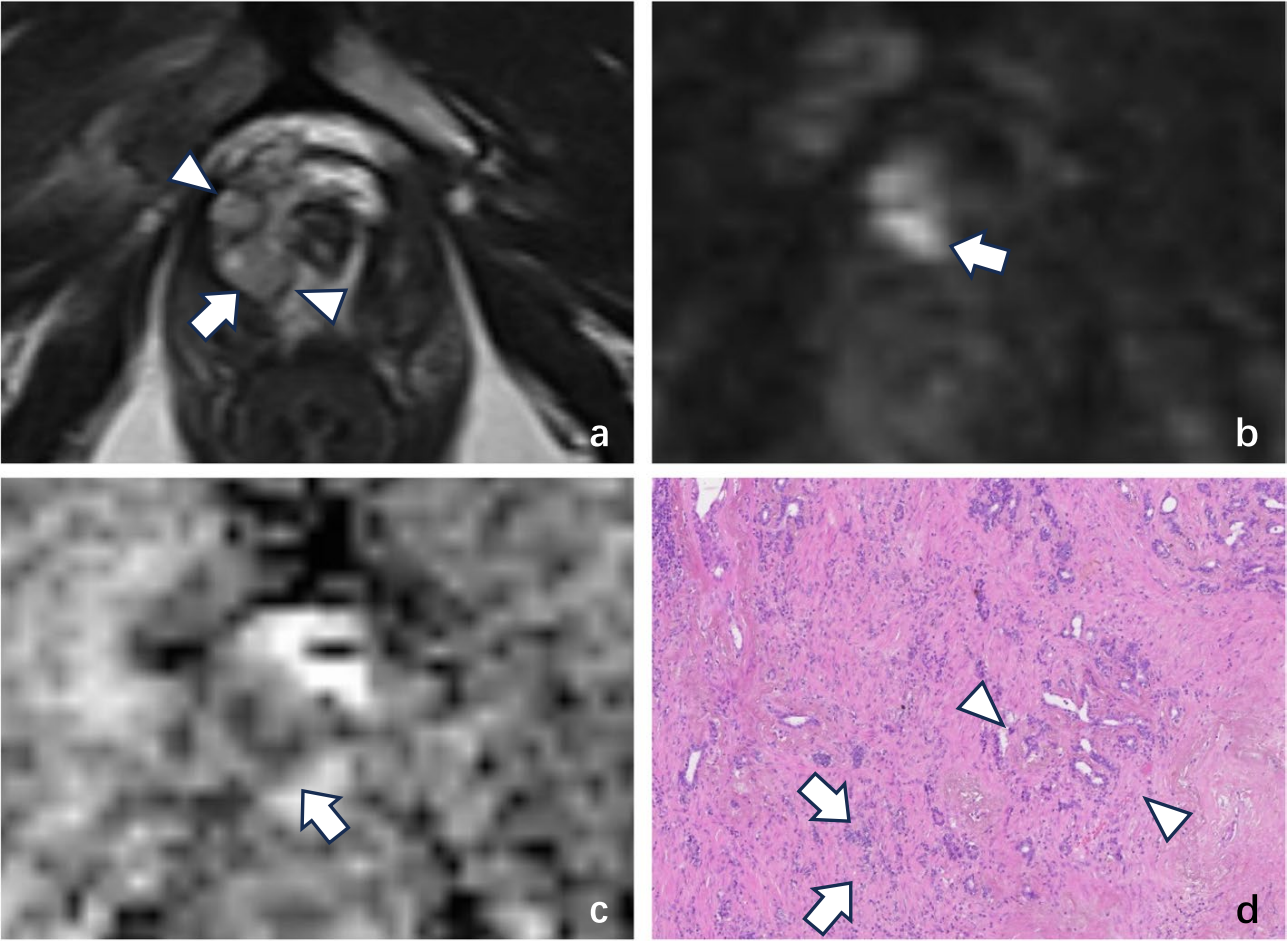
A 67-year-old man with cancer and a right peripheral zone “nodule in nodule” according to T2WI-based PI-RADS v2.1 category 1. **a** Axial T2W MR image demonstrating that the external nodule was completely encapsulated (arrowheads). The inner nodule (white arrow) was characterized as isointense with an obscure margin by both radiologists. On the (**b**) echo-planar DWI (*b* = 1500 mm.^2^/s) map and (**c**) ADC map, the inner nodule (white arrow) was characterized by marked restriction diffusion (DWI score of 4) by the two radiologists. **d** Prostate biopsy histopathological imaging of the “nodule in nodule” yielded a Gleason score of 4 + 5 for prostate acinar cell carcinoma. The image shows that the structure of some glandular cavities has almost completely disappeared, with only occasional glandular cavities visible. Epithelial cells can form solid patches or stripes or can infiltrate the stroma as single cells (arrowheads); additionally, they can undergo partial glandular fusion and sieve-like, glomerular-like or nonglandular luminal structures (arrow) (HE × 100)

**Fig. 4:**
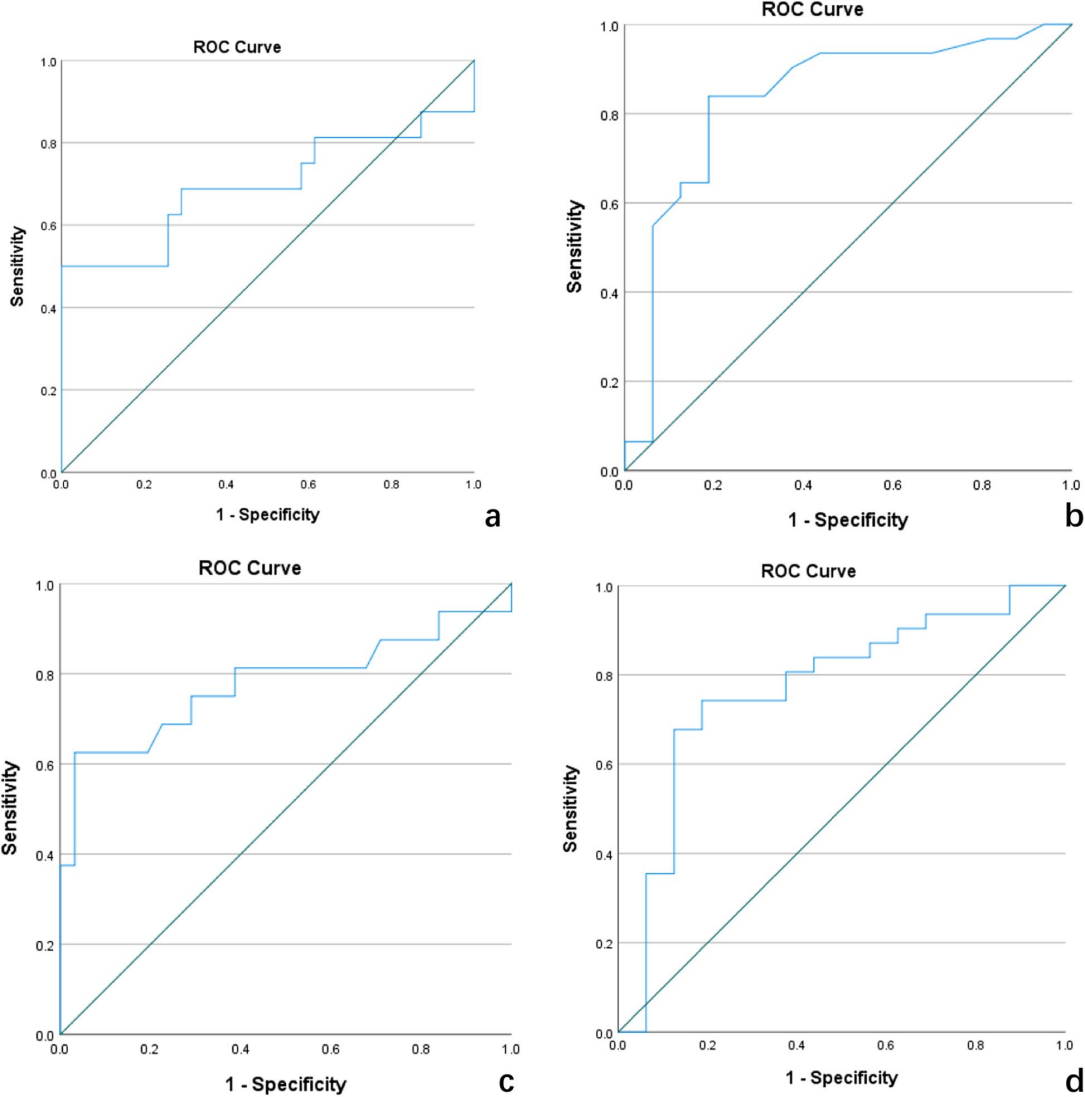
Receiver operating characteristic (ROC) curves of various indices for diagnosing csPCa. **a**, **b**, **c** and **d** show the ROC curves of total PSA, free/total PSA, PSAD, and prostate gland volume for diagnosing csPCa, respectively. **a** Total PSA. **b** f/t PSA. **c** PSAD. **d** Prostate gland volume

A total of 270 nodules were detected in 47 patients (16 with csPCa and 31 without csPCa). Among them, 151 were “nodule in nodule,” including 106 nodules in the TZ and 45 in the PZ. There was a significant difference between the long axis diameters of nodules that did not contain nodules (12.38 ± 4.99 mm) and those that did (15.74 ± 6.57 mm) (*p* < 0.001). Among the “nodule in nodule,” 17 were csPCa lesions that had been identified by pathology, including 11 located in the TZ (Fig. 2) and 6 located in the PZ (Fig. 3). In addition, 12 nodules with more than 2 inner nodules were found simultaneously (9 located in the TZ [all benign prostatic hyperplasia (BPH)]) and 3 located in the PZ [2 PCa and 1 BPH]). The basic data of the patients are shown in Table 2. There was no significant difference in age between csPCa patients (74.18 ± 7.80 years) and non-csPCa patients (74.35 ± 9.84 years; *p* = 0.953). However, there were significant differences in total prostate-specific antigen (tPSA) (csPCa: 180.85 ± 150.14 ng/mL, BPH: 18.57 ± 15.52 ng/mL, *p* = 0.001), free/total PSA (f/tPSA) (csPCa: 0.098 ± 0.071, BPH: 0.197 ± 0.101, *p* < 0.001), PSA density (PSAD) (csPCa: 2.58 ± 2.18, BPH: 0.24 ± 0.17, *p* = 0.001), and PGV (csPCa: 49.05 ± 44.80 mm^3^, BPH: 74.37 ± 35.37 mm^3^, *p* = 0.039) between csPCa patients and BPH patients with “nodule in nodule.” The areas under the receiver operating characteristic (ROC) curves for tPSA, f/t PSA, PSAD, and PGV were 0.696, 0.835, 0.778, and 0.770, respectively (Fig. 4). The corresponding sensitivity and specificity were 68.8%, 83.9%, 62.5%, and 74.2% and 29.0%, 18.8%, 3.2%, and 18.8%, respectively. The cutoff values corresponding to the maximum Youden’s *J* statistic were 18.01 ng/mL, 0.115, 0.57, and 47.14 mm^3^, respectively.

**Table 2:** Patient characteristics. Values are given as the mean ± standard deviation [range] for continuous variables and absolute frequency (relative frequency) for biopsy results

		Nodule in nodule		*p*
PCa		Yes (*n* = 17)	No (*n* = 134)	
Location (*n* = 151)	Transition zone	7.28% (11/151)	62.91% (95/151)	
	Peripheral zone	3.31% (5/151)	26.49% (40/151)	
Age (*n* = 47) (years)		74.18 ± 7.80	74.35 ± 9.84	0.953
Total PSA (*n* = 47) (ng/mL)		180.85 ± 150.14	18.57 ± 15.52	0.001
Free/total PSA (*n* = 47)		0.098 ± 0.071	0.197 ± 0.101	< 0.001
PSAD (*n* = 47)		2.58 ± 2.18	0.24 ± 0.17	0.001
Prostate volume (*n* = 47) (mm^3^)		49.05 ± 44.80	74.37 ± 35.37	0.039

*PSA *prostate-specific antigen, *PSAD *prostate-specific antigen density

**Fig. 5:**
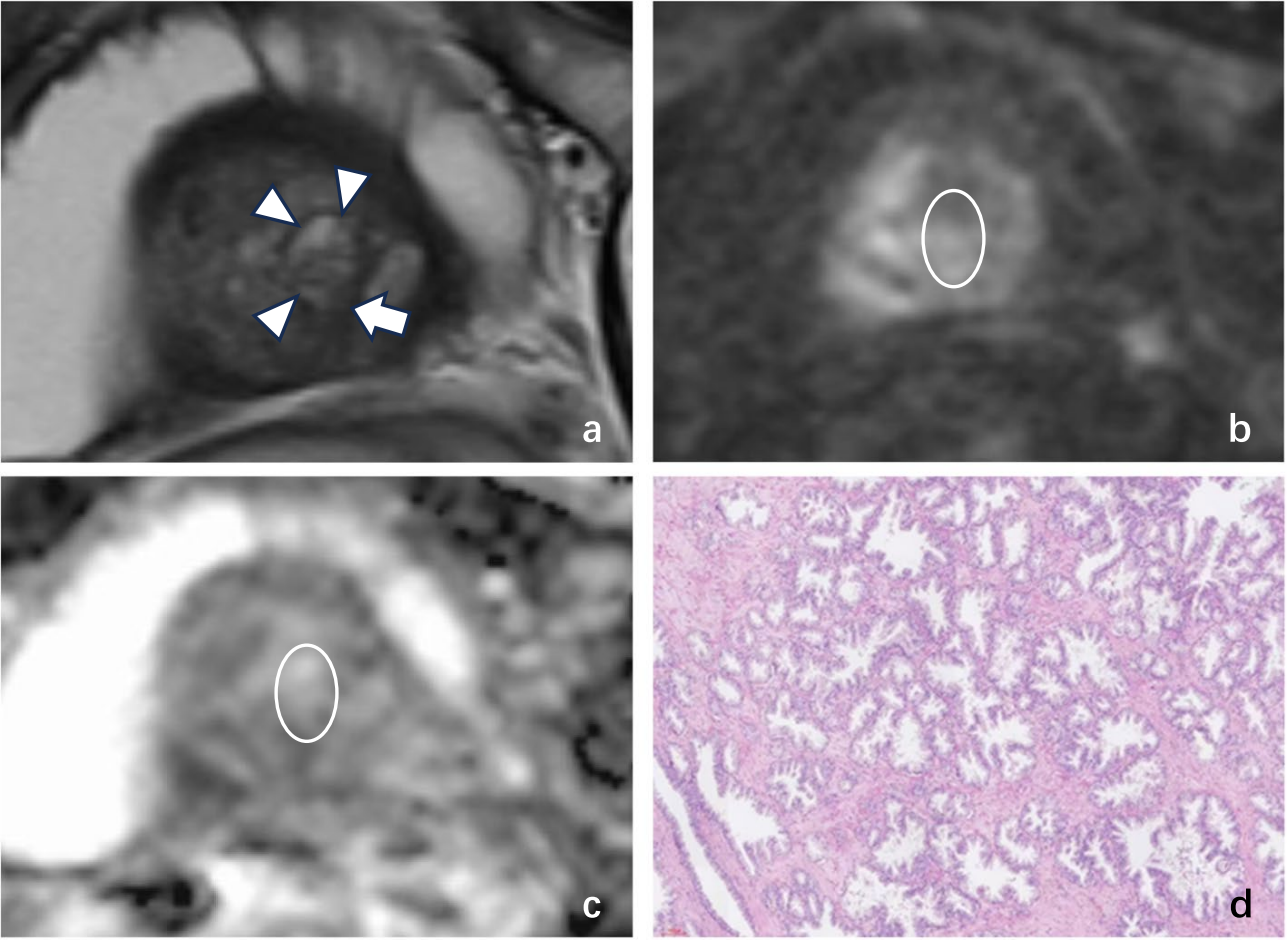
A 71-year-old man with a “nodule in nodule” of PI-RADS V2.1 category 1 in the transition zone without cancer. **a** Axial T2W MR image demonstrating a completely encapsulated of external nodule (arrowheads) with “cystic changes.” The inner nodule (white arrow) was characterized as completely encapsulated by both radiologists. **b** Echo-planar DWI (*b* = 1500 mm.^2^/s) and (**c**) ADC map. The presence of an inner nodule (white circular region of interest) was not associated with marked restricted diffusion (DWI-based score < 3) according to the two radiologists. No cancer was associated with a “nodule in nodule” at biopsy. **d** Prostate biopsy histopathological imaging of the “nodule in nodule” identified that the contour of the prostate gland lumen was undulating, with clustered and papillary folds. Highly secretory epithelial cells had lightly stained and transparent cytoplasm and uniform circular or oval nuclei but no obvious nucleoli. Basal cells can be seen at low magnification (HE × 100)

Radiologists 1 (R1) and 2 (R2) identified 151 and 153 “nodule in nodule,” respectively, with T2WI-based PI-RADS v2.1 scores of 1 in 47 participants (Figs. 2, 3, and 5). For R1 and R2, 7.28% (11/151) and 7.19% (11/153), respectively, of the “nodule in nodule” confirmed as csPCa were located in the TZ (Fig. 2), while 3.97% (6/151) and 3.92% (6/153) which with T2WI-based PI-RADS v2.1 scores of 1 but DWI-based PI-RADS v2.1 scores of 4 were located in the PZ (Fig. 3). The interobserver agreement for the detection of “nodule in nodule” confirmed to be csPCa was 0.930 (95% CI = 0.834–1.000). When comparing csPCa and BPH nodules, both “external” (R1: csPCa, 10.21 ± 6.66 mm, BPH, 16.29 ± 6.35 mm, *p* < 0.001; R2: csPCa, 10.38 ± 6.79 mm, BPH, 16.15 ± 6.33 mm, *p* < 0.001) and inner nodules (R1: csPCa, 4.16 ± 2.82 mm, BPH, 7.51 ± 3.45 mm, *p* < 0.001; R2: csPCa, 4.07 ± 2.90 mm, BPH, 7.58 ± 3.45 mm, *p* < 0.001) were smaller in csPCa patients. The ratio of the long axis diameter of the inner nodule to the long axis diameter of the external nodule was lower for csPCa nodules than for BPH nodules (R1: 0.38 ± 0.12, 0.48 ± 0.17, *p* < 0.001; R2: 0.38 ± 0.10, 0.49 ± 0.17, *p* = 0.001) (Table 3).

**Table 3:** Contingency tables for radiologists 1 and 2 in assessing T2W prostate PI-RADS v2.1 category 1 “nodule in nodule” variant and external and inner nodule size and the sensitivity, specificity, AUC values, and cutoff in the diagnosis of csPCa

		Size of external “nodule in nodule” (EX) (mm)	Size of inner “nodule in nodule” (IN) (mm)	IN/EX
Radiologist 1 PCa	Yes	10.21 ± 6.66	4.16 ± 2.82	0.38 ± 0.12
	No	16.29 ± 6.35	7.51 ± 3.45	0.48 ± 0.17
	*p*	< 0.001	< 0.001	0.004
	Sensitivity	80.6%	79.9%	47.0%
	Specificity	35.3%	29.4%	5.9%
	Cutoff value	10.35	4.75	0.48
	AUC	0.763	0.779	0.680
Radiologist 2 PCa	Yes	10.38 ± 6.78	4.07 ± 2.90	0.38 ± 0.10
	No	16.15 ± 6.33	7.58 ± 3.45	0.49 ± 0.17
	*p*	< 0.001	< 0.001	0.001
	Sensitivity	96.4%	71.5%	51.3%
	Specificity	50.0%	25.0%	6.3%
	Cut-off value	6.4	5.55	0.48
	AUC	0.750	0.789	0.698

R1 detected 17 csPCa “nodule in nodule” on mpMRI, 10.60% (16/151) of which were given a DWI PI-RADS v2.1 score of 4, and 0.66% (1/151) of which were given a score of 3. A total of 27.15% (41/151), 43.71% (66/151), 12.58% (19/151), 3.97% (6/151), and 1.32% (2/151) of the BPH lesions were given scores of 1, 2, 3, 4, and 5, respectively. On T2W images, 3.31% (5/151), 3.31% (5/151), 1.99% (3/151), and 2.65% (4/151) of the “nodule in nodule” were hyperintense, isointense, hypointense, and markedly hypointense, respectively, among the pathologically confirmed csPCa nodules, and 20.53% (31/151), 23.84% (36/151), 35.10% (53/151), and 9.27% (14/151) were hyperintense, isointense, hypointense, and markedly hypointense, respectively, among the BPH nodules. The proportions of “nodule in nodule” classified as encapsulated, circumscribed, or atypical nodules (partially or completely absent capsule) and with an obscured margins were 0.00% (0/151), 0.00% (0/151), 5.96% (9/151), and 5.30% (8/151) among pathologically confirmed csPCa lesions and 27.15% (41/151), 27.15% (41/151), 7.95% (12/151), and 26.49% (40/151), respectively, among BPH nodules. The proportions of “nodule in nodule” that had common morphologies including round, oval, lenticular, lobulated, water-drop-shaped, wedge-shaped, linear, and irregular were 0.66% (1/151), 1.99% (3/151), 1.99% (3/151), 1.32% (2/151), 1.99% (3/151), 1.32% (2/151), 0.00% (0/151), and 1.99% (3/151), respectively, among csPCa nodules and 23.18% (35/151), 25.17% (38/151), 4.64% (7/151), 5.30% (8/151), 4.64% (7/151), 3.31% (5/151), 5.30% (8/151), and 17.22% (26/151), respectively, among BPH nodules. For R2, all 17 csPCa “nodule in nodule” were given a DWI score of 4, while among the BPH nodules, 25.49% (39/153), 46.41% (71/153), 10.46% (16/153), 5.23% (8/153), and 1.31% (2/153) had DWI scores of 1, 2, 3, 4, and 5, respectively. On T2WI, 3.27% (5/153), 3.92% (6/153), 1.96% (3/153), and 1.31% (2/153) of the “nodule in nodule” were hyperintense, isointense, hypointense, and markedly hypointense, respectively, among the pathologically confirmed csPCa nodules, and 20.92% (32/153), 22.88% (35/153), 35.95% (55/153), and 9.80% (15/153) were hyperintense, isointense, hypointense, and markedly hypointense, respectively, among the BPH nodules. The proportions of “nodule in nodule” confirmed as encapsulated, circumscribed, or atypical nodules (partially or completely absent capsule) and with an obscured margins were 0.00% (0/153), 0.00% (0/153), 5.88% (9/153), and 4.58% (7/153) among pathologically confirmed csPCa nodules and 27.45% (42/153), 28.10% (43/153), 9.15% (14/153), and 24.84% (38/153), respectively, among BPH nodules. The proportions of “nodule in nodule” with common morphologies including round, oval, lenticular, lobulated, water-drop-shaped, wedge-shaped, linear, and irregular were 0.65% (1/153), 0.65% (1/153), 1.96% (3/153), 1.31% (2/153), 1.31% (2/153), 0.00% (0/153), 0.66% (1/153), and 3.92% (6/153), respectively, for csPCa, and 22.88% (35/153), 22.22% (34/153), 5.88% (9/153), 8.50% (13/153), 3.92% (6/153), 3.27% (5/153), 5.88% (9/153), and 16.99% (26/153), respectively, for BPH (Table 4).

**Table 4:** Imaging features of inner nodule components of “nodule in nodule” variant for radiologists 1 and 2

		Radiologist 1 PCa		Radiologist 2 PCa	
MRI “nodule in nodule” detected	Yes No	Yes 6.30% (17/270) 8.52% (23/270)	No 49.63% (134/270) 35.56% (96/270)	Yes 6.46% (17/263) 8.37% (22/263)	No 51.71% (136/263) 33.46% (88/263)
“Nodule in nodule” detected Location	TZ	7.28% (11/151)	62.91% (95/151)	7.19% (11/153)	65.36% (100/153)
	PZ	3.97% (6/151)	25.83% (39/151)	3.92% (6/153)	23.53% (36/153)
DWI	1	0.00% (0/151)	27.15% (41/151)	0.00% (0/153)	25.49% (39/153)
	2	0.00% (0/151)	43.71% (66/151)	0.00% (0/153)	46.41% (71/153)
	3	0.66% (1/151)	12.58% (19/151)	0.00% (0/153)	10.46% (16/153)
	4	10.60% (16/151)	3.97% (6/151)	11.11% (17/153)	5.23% (8/153)
	5	0.00% (0/151)	1.32% (2/151)	0.00% (0/153)	1.31% (2/153)
T2WI features	Hyperintense	3.31% (5/151)	20.53% (31/151)	3.27% (5/153)	20.92% (32/153)
	Isointense	3.31% (5/151)	23.84% (36/151)	3.92% (6/153)	22.88% (35/153)
	Hypointense	1.99% (3/151)	35.10% (53/151)	1.96% (3/153)	35.95% (55/153)
	Markedly hypointense	2.65% (4/151)	9.27% (14/151)	1.31% (2/153)	9.80% (15/153)
Margin	Encapsulated	0.00% (0/151)	27.15% (41/151)	0.00% (0/153)	27.45% (42/153)
	Circumscribed	0.00% (0/151)	27.15% (41/151)	0.00% (0/153)	28.10% (43/153)
	Atypical nodule	5.96% (9/151)	7.95% (12/151)	5.88% (9/153)	9.15% (14/153)
	Obscured	5.30% (8/151)	26.49% (40/151)	4.58% (7/153)	24.84% (38/153)
Shape	Round	0.66% (1/151)	23.18% (35/151)	0.65% (1/153)	22.88% (35/153)
	Oval	1.99% (3/151)	25.17% (38/151)	0.65% (1/153)	22.22% (34/153)
	Lenticular	1.99% (3/151)	4.64% (7/151)	1.96% (3/153)	5.88% (9/153)
	Lobulated	1.32% (2/151)	5.30% (8/151)	1.31% (2/153)	8.50% (13/153)
	Water-drop-shaped	1.99% (3/151)	4.64% (7/151)	1.31% (2/153)	3.92% (6/153)
	Wedge-shaped	1.32% (2/151)	3.31% (5/151)	0.00% (0/153)	3.27% (5/153)
	Linear	0.00% (0/151)	5.30% (8/151)	0.66% (1/153)	5.88% (9/153)
	Irregular	1.99% (3/151)	17.22% (26/151)	3.92% (6/153)	16.99% (26/153)

Diffusion-weighted imaging scores obtained according to PI-RADS v2.1. *TZ *transition zone, *PZ *peripheral zone

## Discussion

In clinical practice, we often find that prostate nodules classified as T2WI-based PI-RADS v2.1 scores of 1 exhibit “nodule in nodule” variants that are inconsistent with the morphology of the external nodules. In this study, for R1, 10.60% (16/151) of pathologically confirmed csPCa “nodule in nodule” had a DWI score of 4, and 0.66% (1/151) had a DWI score of 3. Furthermore, 5.96% (9/151) of the inner nodules had incomplete capsulation on T2W images, and 5.30% (8/151) of the nodules had obscured margins (Fig. 2). For R2, 11.11% (17/153) of the “nodule in nodule” confirmed by pathology as csPCa had a DWI score of 4. Furthermore, 5.88% (9/153) of the inner nodules had incomplete capsules on T2W images, and 4.58% (7/153) of the nodules had obscured margins. According to recent studies [21, 22], if PI-RADS v2.1 scoring is performed separately for inner nodules, the inner nodules confirmed by pathology as csPCa in this study with incomplete capsulation or obscured margins on T2WI can be characterized as PI-RADS v2.1 category 2. When the DWI score was ≥ 4 points, the nodule was upgraded to PI-RADS v2.1 category 3 [23]. Lim CS et al. [12] suggested that if the inner nodule of the “nodule in nodule” has incomplete capsulation and apparent restricted diffusion, it should be classified as an atypical nodule, and its PI-RADS v2.1 category should be upgraded to 3. Rudolph MM et al. [15] reported that an obscured margin indicated PCa positivity in 37.6% (35/93) and 32.8% (21/64) of lesions in the PZ and TZ, respectively. Additionally, combining obscured margins with DWI hyperintensity of prostate nodules yielded a positive predictive value of 41.5% (17/41). Based on these findings, we propose that for nodules in the TZ classified as PI-RADS v2.1 category 1, if the inner nodule capsulation is incomplete or the margin is obscured on T2WI and the combined DWI score is ≥ 4, the PI-RADS v2.1 score of the nodule can be upgraded to 3. For nodules with T2WI -based PI-RADS v2.1 scores of 1 in the PZ, the result should comply with DWI-based PI-RADS v2.1 scores. This further confirms the validity of the PI-RADS v2.1 for evaluating prostate nodules in the PZ. Regarding the T2WI signal features and morphology of “nodule in nodule,” there was no significant difference between the csPCa and the non-csPCa patients.

This study showed that the long axis diameters of nodules that contained inner nodules were greater than those of nodules that did not. Due to the diversity of morphology, margins, and signals of prostate “nodule in nodule,” the long axis diameter of the nodules may help us determine the presence of “nodule in nodule.” This requires further validation with a larger sample. Moreover, although both csPCa and non-csPCa patients have larger external nodule diameters than inner nodule diameters, and the ROCs of the long axis diameters of external nodules and inner nodules and their ratio suggest differential diagnostic efficacy for csPCa and non-csPCa, considering the low specificity of these three indicators they still cannot be considered suitable for distinguishing benign and malignant prostate nodules.

Pathologically, patients identified as having csPCa with “nodule in nodule” had higher tPSA and PSAD levels than did non-csPCa “nodule in nodule” patients, while the f/tPSA level was lower in the former. Research by Wei XT’s [24] revealed that a PSAD ≤ 0.33 ng/mL/mL has great differential diagnostic efficacy for csPCa and non-csPCa. The results of this study (cutoff = 0.57 ng/mL/mL) are consistent with those of Wei’s study. Although the PSAD and f/tPSA have good differential diagnostic efficacy and sensitivity for csPCa and non-csPCa, their specificity is relatively low. Wen et al. [25] showed that PSAD is an independent predictor of PCa (PZ: OR = 37.66, 95% CI = 3.3–429.1, *p* = 0.002; TZ: OR = 14.57, 95% CI = 4.64–45.76, *p* < 0.001). However, PSA and PGV were not investigated thoroughly because they are strongly correlated with PSAD. Wang et al. [26] reached the same conclusion as Wen, that is, PSAD is an independent predictor of csPCa (OR = 594.440, 95% CI = 11.395–31,010.36, *p* = 0.002). In contrast, however, they found that f/t PSA was not an independent predictor of csPCa (OR = 0.245, 95% CI = 0.000–190.115, *p* = 0.678). Therefore, we believe that for nodules classified as PI-RADS v2.1 category 1, PSAD is a suitable reference value that can be considered a high-risk factor for csPCa but cannot be used as a diagnostic tool.

There are some shortcomings in our study. The small sample size is a significant drawback; although many nodules were included, the number of nodules confirmed by pathology as csPCa was relatively small. In future studies, it will be necessary to increase the sample size to further verify the related findings. Additionally, the pathological results of the prostate nodules in this study were all derived from biopsy; it is possible that some diagnoses of csPCa were missed.

In conclusion, a prostate nodule with a PI-RADS v2.1 category 1 in the TZ should be carefully monitored for the possibility of malignancy when it contains an inner nodule showing incomplete capsulation or an obscured margin on T2WI and a DWI score ≥ 4. In this case, the PI-RADS v2.1 category 1 lesions in the TZ were updated to category 3. A PSA level ≥ 18.01 ng/mL or a PSAD ≥ 0.57 are high-risk factors for csPCa.

## Data Availability

All authors make sure that all data and materials as well as software application or custom code support their published claims and comply with field standards.

## Abbreviations

BPH: Benign prostatic hyperplasia
csPCa: Clinically significant prostate cancer
DWI: Diffusion-weighted imaging
f/tPSA: Free/total prostate-specific antigen
FOV: Field of view
fPSA: Free prostate-specific antigen
ICC: Intraclass correlation coefficient
mpMRI: Multiparametric MRI
PGV: Prostate gland volume
PI-RADS: Prostate Imaging and Reporting Data System
PSA: Prostate-specific antigen
PSAD: Prostate-specific antigen density
PZ: Peripheral zone
ROC: Receiver operating characteristic
T2WI: T2-weighted imaging
tPSA: Total prostate-specific antigen
TZ: Transition zone
